# CRISPR/CAS9-mediated amino acid substitution reveals phosphorylation residues of RSPH6A are not essential for male fertility in mice^†^

**DOI:** 10.1093/biolre/ioaa161

**Published:** 2020-09-09

**Authors:** Haruhiko Miyata, Ferheen Abbasi, Pablo E Visconti, Masahito Ikawa

**Affiliations:** Department of Experimental Genome Research, Research Institute for Microbial Diseases, Osaka University, Suita, Osaka, Japan; Department of Experimental Genome Research, Research Institute for Microbial Diseases, Osaka University, Suita, Osaka, Japan; Department of Veterinary and Animal Sciences, Integrated Sciences Building, University of Massachusetts Amherst, Amherst, MA, USA; Department of Experimental Genome Research, Research Institute for Microbial Diseases, Osaka University, Suita, Osaka, Japan; The Institute of Medical Science, The University of Tokyo, Minato-ku, Tokyo, Japan

**Keywords:** genome editing, radial spoke, sperm capacitation, sperm motility

Dear Editor,

Capacitation, the physiological changes that occur in mammalian spermatozoa in order to gain the ability to fertilize the egg, is an event that occurs only after spending several hours in the female reproductive tract. Although it is well known that phosphorylation events that start with protein kinase A (PKA) are essential for capacitation [1], downstream phosphorylated molecules and their functions are not well understood. Using phospho-proteomic and subsequent biochemical analyses, we previously identified phosphorylation residues (S17 and S20) of radial spoke head 6 homolog A (RSPH6A) that were associated with capacitation [2]. RSPH6A is an evolutionarily conserved and testis-enriched protein, and thought to be a component of the radial spokes in the flagellar axoneme [2–4]. In a previous study, we generated *Rsph6a* knock-out (KO) mice to analyze its function; however, because *Rsph6a* KO spermatozoa exhibited abnormal flagellar formation [4], this hampered the analysis of RSPH6A in sperm capacitation. To overcome this, we substituted two serine residues of mouse RSPH6A to alanine using the CRISPR/CAS9 system.

We electroporated a crRNA/tracrRNA/Cas9 ribonucleoprotein and a single-stranded oligonucleotide containing the S17A and S20A mutations into the fertilized eggs (Figure 1A and Supplementary Methods). In addition to amino acid substitutions, several silent mutations were introduced to prevent the mutated region from being recognized and recut by the ribonucleoprotein (Figure 1A). Of the 97 fertilized oocytes that were electroporated, 62 two-cell embryos were transplanted into the oviducts of three pseudopregnant female mice. Ten pups were born and one of the ten pups had the intended mutation. Subsequent breeding resulted in a knock-in (KI) mouse with the S17A and S20A mutations, which was confirmed by PCR and NarI digestion (Figure 1B) as well as sequencing analysis (Figure 1C). Although point mutations could lead to decreased or diminished amounts of targeted protein [5, 6], a comparable amount of RSPH6A in spermatozoa collected from the cauda epididymis was observed in *Rsph6a^KI/KI^* mice with immunoblotting (Figure 1D).

**Figure 1:**
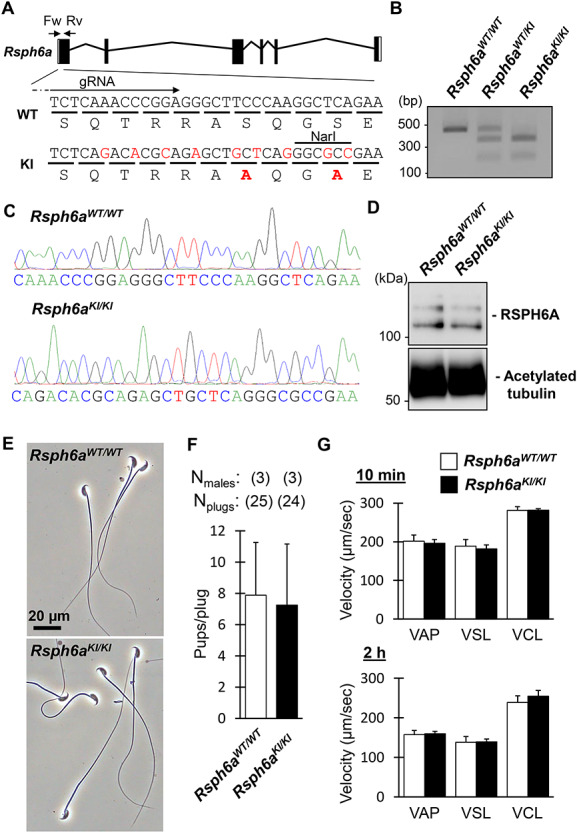
Phosphorylation of RSPH6A S17 and S20 is not essential for male fertility. **A.** CRISPR/Cas9 targeting scheme. gRNA was designed to target Exon1. Primers (Fw, Rv) used for genotyping are shown. Mutated nucleotides and amino acids are written in red. **B.** Genotyping of *Rsph6a^KI/KI^* mice using NarI. **C.** Wave pattern sequence of *Rsph6a* confirming the mutated allele. **D.** Protein expression of RSPH6A in cauda epididymal spermatozoa. Acetylated tubulin as a loading control. **E.** Observation of spermatozoa obtained from cauda epididymis. **F.** The fertility of *Rsph6a^KI/KI^* male mice. **G.** VAP (average path velocity), VSL (straight line velocity) and VCL (curvilinear velocity) were analyzed 10 min (non-capacitated) and 2 h (capacitated) after sperm incubation; *n* = 4 males each for *Rsph6a^WT/WT^* and *Rsph6a^KI/KI^* mice.

In contrast to impaired flagellar formation observed in *Rsph6a* KO spermatozoa [4], no abnormal morphology was observed with phase-contrast microscopy in spermatozoa collected from the cauda epididymis of *Rsph6a^KI/KI^* mice (Figure 1E). We then mated *Rsph6a^KI/KI^* males with three wild-type females for 2 months. No significant difference was observed between *Rsph6a^WT/WT^* and *Rsph6a^KI/KI^* mice (Figure 1F). Next, we analyzed sperm motility with a computer-assisted sperm analysis system. The percentage of motile spermatozoa was comparable between *Rsph6a^WT/WT^* mice (10 min: 87.9 ± 4.4%, 2 h: 75.1 ± 11.2%, *n* = 4 males) and *Rsph6a^KI/KI^* mice (10 min: 84.5 ± 1.5%, 2 h: 70.2 ± 5.0%, *n* = 4 males). Furthermore, no significant differences were observed in velocity parameters of non-capacitated (10 min) and capacitated (2 h) spermatozoa between *Rsph6a^WT/WT^* and *Rsph6a^KI/KI^* mice (Figure 1G). Taken together, these results indicate that S17 and S20 phosphorylation are not essential for sperm formation, motility, and male fertility.

Super-resolution imaging with stochastic optical reconstruction microscopy suggests that the catalytic PKA subunit can be localized in the sperm axoneme [7]. RSPH6A localized in the axoneme was identified as a PKA substrate candidate using phospho-proteomic analysis [2]; however, this study reveals that S17 and S20 of RSPH6A are not essential for male fertility. As is the case for RSPH6A [4], disruption of axonemal proteins often leads to impaired flagellar formation [8], which makes it challenging to analyze their function in mature spermatozoa using a straight KO approach. To overcome this problem, amino acid substitution can be introduced with the CRISPR/CAS9 system, which can be conducted more easily in a shorter period of time than the conventional method that uses homologous recombination in embryonic stem cells [5]. In addition, CRISPR/CAS9 enables us to manipulate the genome without introducing selection cassettes such as drug resistance. Considering the difficulty in manipulating the genome of spermatozoa and eggs in vitro, CRISPR/CAS9-mediated amino acid substitution in an organismal level will be the future of the reproduction field and could uncover the hidden mysteries of PKA downstream effectors in sperm capacitation.

## Author Contributions

H.M, P.E.V, and M.I designed research; H.M, and F.A performed research; H.M, F.A, P.E.V, and M.I analyzed data; and H.M, F.A, P.E.V, and M.I wrote the paper.

## Supplementary Material

Miyata_et_al_Supplementary_Methods_ioaa161

## References

[1] Nolan MA, Babcock DF, Wennemuth G, Brown W, Burton KA, Mcknight GS Sperm-specific protein kinase a catalytic subunit Cα2 orchestrates cAMP signaling for male fertility. Proc Natl Acad Sci USA 2004; 101:13483–13488. 15340140 10.1073/pnas.0405580101 PMC518783

[2] Paudel B, Gervasi MG, Porambo J, Caraballo DA, Tourzani DA, Mager J, Platt MD, Salicioni AM, Visconti PE Sperm capacitation is associated with phosphorylation of the testis-specific radial spoke protein Rsph6a. Biol Reprod 2019; 100:440–454. 30239614 10.1093/biolre/ioy202 PMC6378865

[3] Curry AM, Williams BD, Rosenbaum JL Sequence analysis reveals homology between two proteins of the flagellar radial spoke. Mol Cell Biol 1992; 12:3967–3977. 1508197 10.1128/mcb.12.9.3967-3977.1992 PMC360281

[4] Abbasi F, Miyata H, Shimada K, Morohoshi A, Nozawa K, Matsumura T, Xu Z, Pratiwi P, Ikawa M RSPH6A is required for sperm flagellum formation and male fertility in mice. J Cell Sci 2018; 131:jcs221648. 30185526 10.1242/jcs.221648 PMC6198453

[5] Abbasi F, Miyata H, Ikawa M Revolutionizing male fertility factor research in mice by using the genome editing tool CRISPR/Cas9. Reprod Med Biol 2017; 17:3–10. 29371815 10.1002/rmb2.12067 PMC5768971

[6] Nozawa K, Satouh Y, Fujimoto T, Oji A, Ikawa M Sperm-borne phospholipase C zeta-1 ensures monospermic fertilization in mice. Sci Rep 2018; 8:1315. 29358633 10.1038/s41598-018-19497-6 PMC5778054

[7] Stival C, Ritagliati C, Xu X, Gervasi MG, Luque GM, Baró Graf C De la Vega-Beltrán JL, Torres N, Darszon a, Krapf D, Buffone MG, Visconti PE, et al. Disruption of protein kinase a localization induces acrosomal exocytosis in capacitated mouse sperm. J Biol Chem 2018; 293:9435–9447. 29700114 10.1074/jbc.RA118.002286 PMC6005427

[8] Miyata H, Morohoshi A, Ikawa M Analysis of the sperm flagellar axoneme using gene-modified mice. Exp Anim 2018. doi: 10.1538/expanim.20-0064 Online ahead of print. PMC7677079 32554934

