# Supplementary material for: CRISPR/CAS9-mediated amino acid substitution reveals phosphorylation residues of RSPH6A are not essential for male fertility in mice
Source: Biol Reprod. 2020 Sep 9;103(5):912–4. doi: 10.1093/biolre/ioaa161 (PMC7609874; doi:10.1093/biolre/ioaa161)
Supplement: Miyata_et_al_Supplementary_Methods_ioaa161 [file miyata_et_al_supplementary_methods_ioaa161.docx]

**CRISPR/CAS9-mediated amino acid substitution reveals phosphorylation residues of RSPH6A are not essential for male fertility in mice**

Haruhiko Miyata, Ferheen Abbasi, Pablo E. Visconti, Masahito Ikawa

**Supplementary Methods**

**Materials and Methods**

***Animals***

Mice were purchased from Japan SLC (Shizuoka, Japan) or CLEA Japan (Tokyo, Japan). All animal experiments were approved by the Animal Care and Use Committee of the Research Institute for Microbial Diseases, Osaka University (#Biken-AP-H30-01).

***Egg collection for genome editing***

CARD HyperOva (0.1 mL, Kyudo, Saga, Japan) was injected into the abdominal cavity of B6D2F1 female mice, followed by human chorionic gonadotropin (hCG) (five units, ASKA Pharmaceutical, Tokyo, Japan) with 48 h interval. Superovulated females were mated with B6D2F1 males and fertilized eggs were collected from the oviduct.

***Generation of Rsph6a knock-in mice and genotyping***

Electroporation was performed as described previously [1]. Briefly, crRNA/tracrRNA/Cas9 ribonucleoproteins (40 ng/μl crRNA plus tracrRNA, 100 ng/μl CAS9) and oligonucleotide (200 ng/μl) were electroporated into the fertilized eggs using a super electroporator NEPA21 (NEPA GENE, Chiba, Japan) (poring pulse, voltage: 225 V, pulse width: 2 ms, pulse interval: 50 ms, and number of pulses: +4; transfer pulse, voltage: 20 V, pulse width: 50 ms, pulse interval: 50 ms, and number of pulses: ±5). The gRNA and oligonucleotide sequences are written below. The electroporated eggs were cultivated in potassium simplex optimization medium (KSOM) [2] and the two-cell- embryos were transferred into the oviducts of pseudopregnant ICR females the next day. The pups were genotyped by PCR and subsequent NarI enzyme digestion or sequencing. The primers used for genotyping are written below. *Rsph6a^KI/WT^* mice (B6D2-*Rsph6a^em3Osb^*) are being processed for deposition to the Riken BioResource Center, Japan or Center for Animal Resources and Development (CARD), Kumamoto University, Japan.

gRNA sequence: 5’-TGACCCCTCTCAAACCCGGA-3’

Oligonucleotide sequence:

5’-CACTTTTTCCGAGGCATGGGGGAACCACCGCCCAATCCTGACCCCTCTCAGACAC

GCAGAGCTGCTCAGGGCGCCGAAAGGGCACGGAGTCAAGAGTACTCTCAGCCTCTGTTAACCATCCCAGA-3’.

Primer for genotyping (Fw): 5’-GGTAAAGTAACGGTAGGCAGGCAGGG-3’

Primer for genotyping (Rv): 5’-CTGCTCCATCATCCTGCTATCATCCAGG-3’

***Immunoblotting***

Spermatozoa were homogenized in a lysis buffer containing 6 M urea, 2 M thiourea, and 2% sodium deoxycholate, and then centrifuged at 15,000 g for 15 min to collect supernatant samples. The samples were subjected to SDS-PAGE followed by immunoblotting as described previously [1]. Blots were blocked with 10% skim milk, incubated with primary antibodies overnight at 4 ℃, and incubated with secondary antibodies conjugated to horseradish peroxidase (1:10,000) (#111-036-045 or #115-036-062, Jackson ImmunoResearch, PA, USA) for 2 h at room temperature. Primary antibodies used were rabbit anti-RSPH6A (1:1000) [1] and mouse anti-acetylated tubulin (1:1000) (#T7451, Sigma-Aldrich, MO, USA). Signals were detected by an ECL western blotting detection kit (GE Healthcare, Little Chalfont, UK).

***Mating exam***

Three sexually mature male mice were caged with three 8-week-old B6D2F1 female mice each for 2 months. Plugs were checked every morning and the number of pups was counted on the day of birth.

***Sperm morphology and motility***

Spermatozoa were collected from the cauda epididymis and suspended in a 100 µl drop of TYH medium [3]. Sperm morphology was observed with a BX-53 microscope (Olympus, Tokyo, Japan). Sperm motility was analyzed with the CEROS II sperm analysis system (software version 1.4; Hamilton Thorne Biosciences, MA, USA) 10 min and 2 h after sperm incubation.

**Supplementary Methods References**

1. Abbasi F, Miyata H, Shimada K, Morohoshi A, Nozawa K, Matsumura T, Xu Z, Pratiwi P, Ikawa M. RSPH6A is required for sperm flagellum formation and male fertility in mice. J Cell Sci 2018; 131:jcs221648.
2. Ho Y, Wigglesworth K, Eppig JJ, Schultz RM. Preimplantation development of mouse embryos in KSOM: augmentation by amino acids and analysis of gene expression. Mol Repod Dev 1995; 41:232-238.
3. Muro Y, Hasuwa H, Isotani A, Miyata H, Yamagata K, Ikawa M, Yanagimachi R, Okabe M. Behavior of Mouse Spermatozoa in the Female Reproductive Tract from Soon after Mating to the Beginning of Fertilization. Biol Reprod 2016; 94:1-4.
